# Butyrate acts through HDAC inhibition to enhance aryl hydrocarbon receptor activation by gut microbiota-derived ligands

**DOI:** 10.1080/19490976.2022.2105637

**Published:** 2022-07-27

**Authors:** Morgane Modoux, Nathalie Rolhion, Jeremie H. Lefevre, Cyriane Oeuvray, Petr Nádvorník, Peter Illes, Patrick Emond, Yann Parc, Sridhar Mani, Zdenek Dvorak, Harry Sokol

**Affiliations:** a Sorbonne Université, INSERM, Centre de Recherche Saint-Antoine, CRSA, AP-HP, Saint Antoine Hospital, Gastroenterology Department, Paris, France; b Paris Centre for Microbiome Medicine (PaCeMM) FHU, Paris, France; c Sorbonne Université, Department of Digestive Surgery, AP-HP, Hôpital Saint Antoine, Paris, France; d Departments of Cell Biology and Genetics, Palacký University, Olomouc, Czech Republic; e UMR 1253, iBrain, Université de Tours, Inserm, Tours, Centre-Val de Loire, France; f Departments of Molecular Pharmacology, Genetics and Medicine, Albert Einstein College of Medicine, Bronx, NY, USA; g INRAe, UMR1319 Micalis & AgroParisTech, Jouy en Josas, France

**Keywords:** Microbiota, AhR, butyrate, FICZ, HDAC, *CYP1A1*, metabolites, SCFAs, tryptophan

## Abstract

Aryl hydrocarbon receptor (AhR) is a critical player in the crosstalk between the gut microbiota and its host. However, factors regulating AhR within the gut, which is a complex metabolomic environment, are poorly understood. This study investigates the effect of a combination of metabolites on the activation mechanism of AhR. AhR activity was evaluated using both a luciferase reporter system and mRNA levels of AhR target genes on human cell lines and human colonic explants. AhR activation was studied by radioligand-binding assay, nuclear translocation of AhR by immuofluorescence and protein co-immunoprecipitation of AhR with ARNT. Indirect activation of AhR was evaluated using several tests and inhibitors. The promoter of the target gene *CYP1A1* was studied both by chromatin immunoprecipitation and by using an histone deacetylase HDAC inhibitor (iHDAC). Short-chain fatty acids, and butyrate in particular, enhance AhR activity mediated by endogenous tryptophan metabolites without binding to the receptor. This effect was confirmed in human intestinal explants and did not rely on activation of receptors targeted by SCFAs, inhibition of AhR degradation or clearance of its ligands. Butyrate acted directly on AhR target gene promoter to reshape chromatin through iHDAC activity. Our findings revealed that butyrate is not an AhR ligand but acts as iHDAC leading to an increase recruitment of AhR to the target gene promoter in the presence of tryptophan-derived AhR agonists. These data contribute to a novel understanding of the complex regulation of AhR activation by gut microbiota-derived metabolites.

## Introduction

The gut microbiota is a complex ecosystem essential for host defense against infection, nutrient metabolism, and tissue repair. It synthesizes metabolites from dietary and host-derived molecules to modulate the host’s metabolism.^1–5^ These metabolites represent a central hub in the host-microbiota crosstalk and are dysregulated in several diseases, including inflammatory bowel diseases (IBD), metabolic syndrome, and neuropsychiatric conditions.^6–8^ The aryl hydrocarbon receptor (AhR) pathway is involved in several metabolic and immune processes, which are vital for intestinal homeostasis, as well as for optimal coexistence of the host and its microbiome.^9^ First characterized as a receptor for environmental toxins, AhR is now recognized as a receptor for many microbial tryptophan (Trp) derivatives and is therefore emerging as an attractive pharmacological target for several conditions such as IBD, celiac disease, metabolic syndrome, liver disease, neurological disease and cancers.^10–15^

After ligand binding, AhR translocates into the nucleus and dimerizes with AhR nuclear translocator (ARNT) before the dimer binds the target genes promoter sequence leading to their transcription. Target genes include cytochromes (CYP; *CYP1A* and *CYP1B*), interleukin *IL22*, and the AhR repressor (*AHRR*). Beyond microbial Trp metabolites, AhR binds many natural or synthetic ligands with a wide range of structural diversity.^16,17^ About 70% of the observed variance in fecal metabolites level is explained by the composition of the microbiota rather than host genetics.^18^ Moreover, experiments in germ-free mice suggest that the abundance of 70% of the gut metabolites in colon content is impacted by the gut microbiota.^19^ Nevertheless, no study has yet systematically investigated the impact of gut-derived microbial metabolites apart from Trp metabolites on AhR activity to date.

Here, following a systematic screen in vitro, we identified that the SCFA butyrate activates the AhR pathway. In further experiments, we demonstrated that butyrate does not bind to AhR but synergizes with known ligands to enhance its activation. This synergistic effect between butyrate and Trp-derived AhR agonist was transposable ex vivo on human intestinal explants. We showed that butyrate acts by inhibiting histone deacetylases (HDAC), thus opening chromatin to increase the access of AhR-ligand complexes to their binding sites in the promoter of AhR target genes. Collectively, our data showed a crucial role of microbiota-derived SCFAs in modulating AhR activation induced by Trp-derived agonists.

## Results

Several gut microbiota-dependent metabolites activate AhR in vitro.

**Figure 1.**
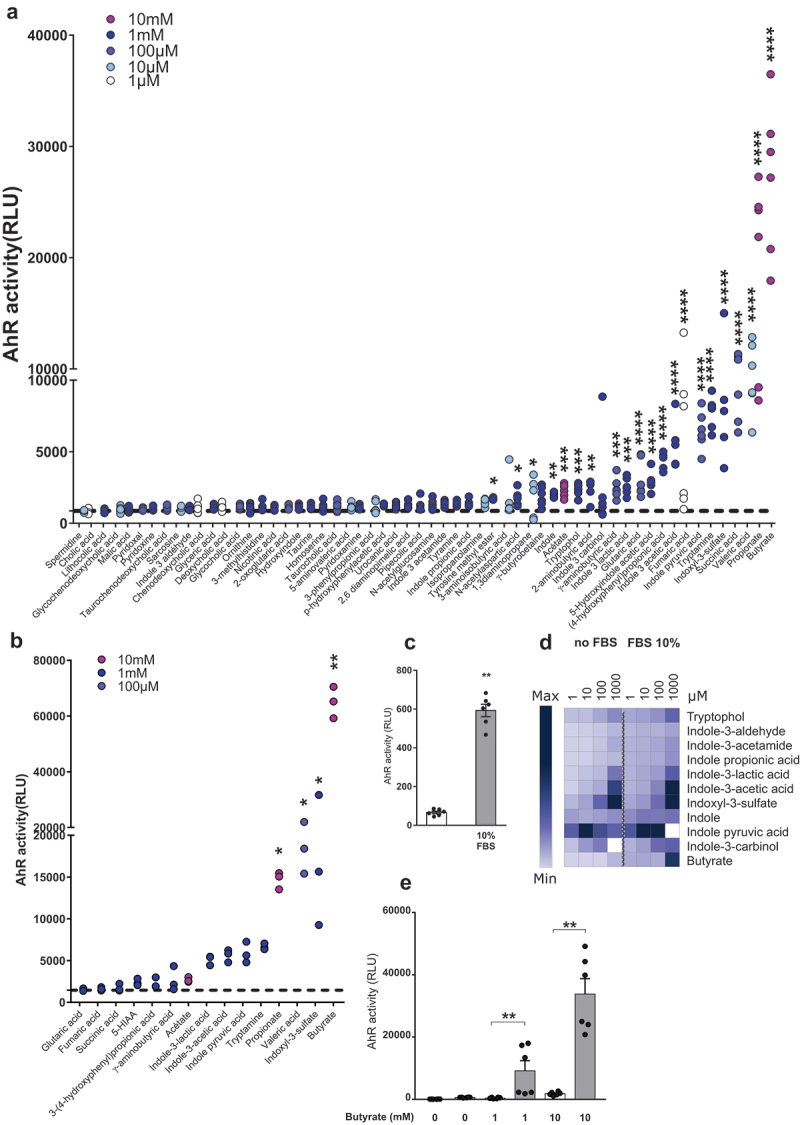
Several gut microbiota-dependent metabolites activate AhR. (a) HepG2^luc^ were treated in triplicate for 24 hours with differents doses of metabolites (1 µM – 10 mM). Results were normalized on the basis of negative luciferase activity of the control (unstimulated cells, dotted line) and cytotoxicity measurement and doses with the highest AhR activity without cell mortality are shown. Data are representative of two independent experiments. (b) HT29^luc^ were treated in triplicate for 24 hours with the metabolites having the highest AhR activity on HepG2^luc^. Doses with the highest AhR activity without cell mortality are shown. (c) AhR activity in HepG2^luc^ without (white) or with 10% FBS (gray) after 24 hours. Cells were treated in triplicate and data are presented as mean ± SEM of two independent experiments. (d) Heat map representation of AhR activation in HepG2^luc^ treated in triplicate with Trp metabolites and SCFAs (1 µM – 1 mM) with or without 10% FBS. Means RLU obtained in two independent experiments were represented and relative color graduation was attributed using Morpheus software (https://software.broadinstitute.org/morpheus). (e) AhR activity obtained in HepG2^luc^ treated in triplicate with butyrate (1 and 10 mM) without (white) or with (gray) 10% FBS. Data are presented as mean ± SEM of two independent experiments. *p < .05; **p < .01; ***p < .001; ****p < .0001.

To get an overall picture of the ability of microbiota-derived metabolites to activate AhR, we screened fifty-eight gut microbiota-dependent metabolites (some of which were identified by Matsumoto and colleagues)^19^ on AhR reporter human liver cell line (HepG2^luc^), which are highly sensitive to AhR agonists. As expected, high AhR agonist activity was found for Trp-derived metabolites, like indoxyl-3 sulfate and tryptamine (Figure 1a). Surprisingly, the highest AhR activation was found for the SCFAs butyrate and propionate. We then evaluated the effect of best hits from this first screen in a human reporter intestinal epithelial cell line (HT29^luc^). Most metabolites activating AhR in HepG2^luc^ were also effective in HT29^luc^ and butyrate was also the most potent on these cells (Figure 1b).

### Butyrate and AhR ligands act synergistically to activate AhR

Serum of humans and mice has been shown to contain several indole compounds activating AhR.^20,21^ Indeed fetal bovine serum (FBS) used for cell culture screens also contains AhR agonists, as demonstrated by its ability to activate HepG2^luc^ cells (Figure 1c). Targeted quantitative metabolomics confirmed the presence of indoles in mouse, human and FBS (Figures S1A and S1B). Most indole derivatives retained AhR activity in the absence of FBS, but not butyrate (Figures 1D and 1e), suggesting that butyrate does not directly activate AhR but might synergize with indoles present in FBS.

**Figure 2.**
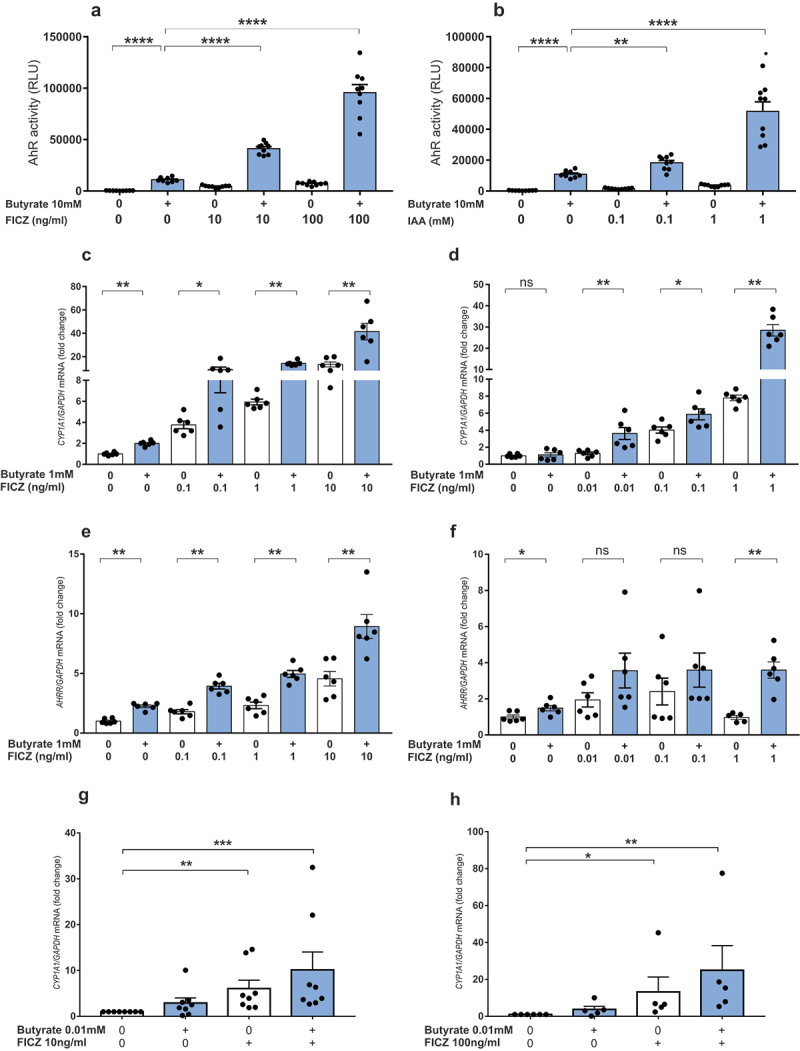
Butyrate and AhR ligands synergize to activate the AhR pathway. (a-b) AhR activity in HepG2^luc^ treated in triplicate with butyrate (10 mM) in combination with FICZ(10 and 100 ng/ml)(a) or IAA (0.1 and 1 mM) (b) in FBS-free media. Data are presented as mean ± SEM of three independent experiments. (c-d) RT-qPCR analysis of *CYP1A1* mRNA levels in HepG2 (c) and HT29 (d) treated with butyrate and/or FICZ for 8 and 6 hours respectively. (e-f) RT-qPCR analysis of *AHRR* mRNA levels in HepG2 (e) and HT29 (f) treated with butyrate and/or FICZ for 8 and 6 hours respectively. Data are presented as mean ± SEM of two independent experiments. (g-h) RT-qPCR analysis of *CYP1A1* mRNA levels in colonic explants treated with butyrate (0.1 and 0.01 mM) in combination with FICZ 10 ng/ml (g) or 100 ng/ml (h) for 24 hours. Data are presented as mean ± SEM and each dot represent a patient. *p < .05, **p < .01, ***p < .001, ****p < .0001.

To confirm this hypothesis, we combined SCFAs with known AhR agonists FICZ and indole acetic acid (IAA) on HepG2^luc^ and HT29^luc^ cells without FBS. We used the microbiota-derived IAA as we previously showed its decrease in IBD^11^ and metabolic syndrome.^14^ FICZ is an endogenous photoproduct of Trp metabolism with a potent AhR-activity.^22^ Butyrate, FICZ and IAA led to significant AhR activation on HepG2^luc^ cells, but synergistically increased when they were combined with butyrate (Figures 2a and 2b). Synergistic effect between SCFAs and FICZ or IAA was also observed for propionate (Figures S2A and S2B) and acetate (Figures S2C and S2D). A similar effect was observed on HT29^luc^ cells with butyrate (Figures S3A and S3B) and propionate (Figures S3C and S3D), but not for acetate, except with the highest dose of FICZ (Figures S3E and S3F). The synergistic effect of combining butyrate with FICZ or IAA on AhR activation was also confirmed on *CYP1A1* (Figures 2c and 2d) and *AHRR* (Figures 2e and 2f) expression on conventional HepG2 and HT29 cell lines. Taken together, these results show that SCFAs, and particularly butyrate, synergize with Trp metabolites to activate AhR. To test the relevance of our results in humans, we treated healthy colonic explants from patients operated for colorectal cancer with butyrate, FICZ or a combination of both. Induction of *CYP1A1* expression by FICZ was increased by co-treatment with butyrate (Figures 2g and 2h), confirming in primary cells the effect observed in cell lines.

### Butyrate does not interact directly with AhR

**Figure 3.**
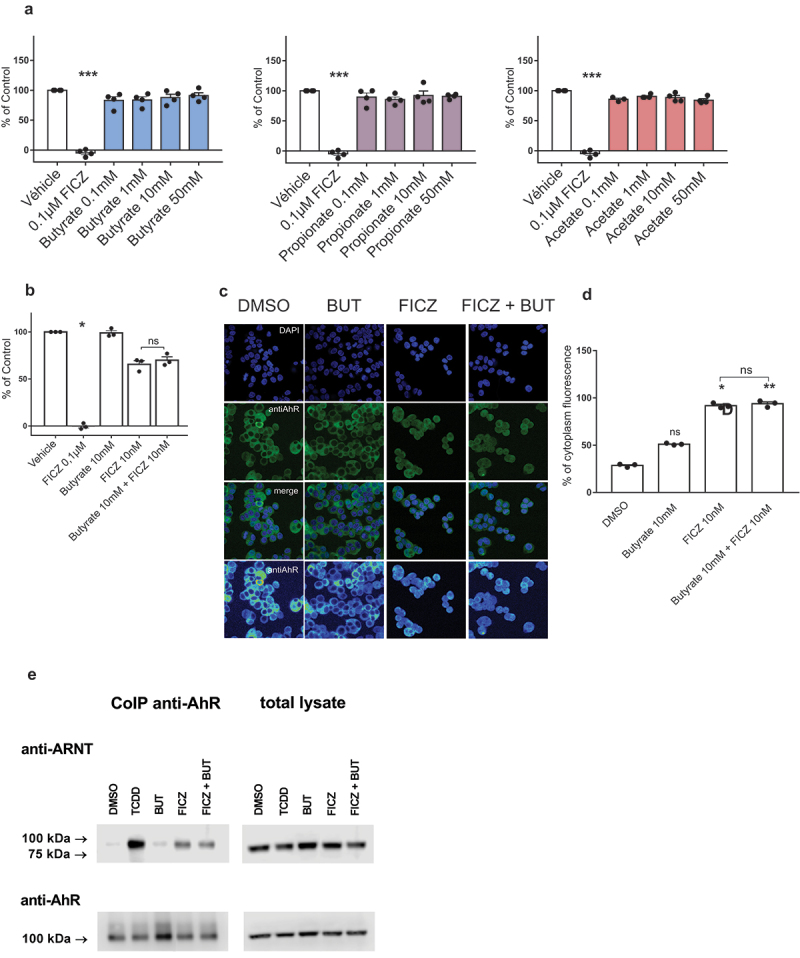
Butyrate is not an AhR ligand. (a-b) Ligand binding to AhR was evaluated for SCFAs (a) and the combination of butyrate with FICZ (b). Data are presented as mean ± SEM of three independent experiments. (c) Nuclear translocation of AhR in LS180 cells treated with vehicle (DMSO), butyrate, FICZ and FICZ combined with butyrate. (d) Semi-quantitative estimation of nuclear translocation was expressed as a percentage of intensity of cytoplasm fluorescence. (e) Formation of AhR-ARNT heterodimers in LS180 treated for 90 min with TCDD, (10 nM), butyrate (10 mM), FICZ (10 nM) and combination of butyrate with FICZ. * p < .05, **p < .01, ***p < .001.

A recent in silico structural modeling study suggested that butyrate might bind to AhR.^17^ We tested the ability of SCFAs to physically bind to AhR using a radio-ligand binding assay in the murine hepatoma cells Hepa1c1c7. While low dose FICZ was highly effective to displace radiolabeled 2,3,7,8-tetrachlorodibenzo-p-dioxine (TCDD) from AhR, even high doses of butyrate, propionate, and acetate failed to do so (Figure 3a). Moreover, adding butyrate to a low dose of FICZ did not increase the displacement of TCDD compared to FICZ alone (Figure 3b). Thus, SCFAs are not AhR ligands.

We then investigated whether butyrate induces AhR nuclear translocation by immunofluorescence assay on human colonic epithelial cell line. FICZ induced nuclear translocation of AhR, but butyrate neither induced nuclear translocation by itself nor increased the nuclear translocation induced by FICZ (Figures 3c and 3d). Therefore, butyrate does not act on AhR nuclear translocation.

To assess if butyrate potentiates the formation of the AhR-ARNT dimer, we performed a co-immunoprecipitation experiment using anti-AHR antibody followed by immunoblotting with ARNT antibody. TCDD and FICZ, but not butyrate, induced the formation of AhR-ARNT heterodimers. Furthermore, adding butyrate to FICZ did not result in any changes in the formation of the AhR-ARNT complex (Figure 3e). Therefore, butyrate does not act on the formation of the AhR-ARNT heterodimer. Taken together, these results show that butyrate does act neither at the ligand-binding level nor at the nuclear translocation and heterodimerization level. We thus hypothesized that butyrate acts downstream of AhR-ARNT formation.

### Butyrate does not act through indirect mechanisms.

The prototypic AhR target gene *CYP1A1* is involved in the metabolism of AhR ligands such as benzo-α-pyrene and FICZ.^22–24^ Thus, CYP1A1 inhibition is an indirect mechanism to activate AhR by interrupting the ligands’ clearance.^22^ We tested the ability of butyrate to inhibit the human recombinant CYP1A1. Unlike the classical CYP1A1 inhibitor alpha naphthoflavone, butyrate did not induce any change in the activity of CYP1A1 compared to control (Figure S4A). Therefore, butyrate does not activate AhR by inhibiting CYP1A1-dependent clearance of ligands.

SCFAs are ligands of several G protein-coupled receptors (GPCRs), but their involvement in AhR activation is controversial.^17,25^ We explored if the GPCRs FFAR2, FFAR3 and HCAR2, known to be activated by SCFAs, were involved in the butyrate-mediated enhancement of AhR activity. We did not find any expression of *FFAR2* and *FFAR3* on both HT29 and HepG2 cells (data not shown), ruling out the implication of these two receptors in butyrate-mediated activation of AhR. *HCAR2* expression was observed in both cell lines and induced by butyrate in HT29 cells (Figure S4B). To explore the role of HCAR2 in the synergistic effect, we treated HepG2^luc^ cells with butyrate or FICZ in the presence or absence of the HCAR2 antagonist mepenzolate bromide (MB). MB impacted neither FICZ nor butyrate combined with FICZ-mediated AhR activity (Figure S4C). HCAR2 is thus not involved in butyrate-mediated activation of AhR.

The ubiquitin-proteasome pathway degrades cellular proteins, including AhR, and inhibition of this pathway leads to nuclear translocation of AhR and nuclear accumulation of active AhR.^26,27^ As butyrate can act as a proteasome inhibitor,^28^ we tested if butyrate induced AhR activation by proteasome inhibition. The proteasome inhibitor MG132 increased neither FICZ-induced AhR activation nor the synergy between butyrate and FICZ (Figure S4D). Surprisingly, proteasome inhibition indeed suppressed the activation of AhR. Collectively, these data show that butyrate acts neither by CYP1A1 inhibition nor by GPCRs-dependent signaling or proteasome inhibition.

### Butyrate acts on AhR via its inhibitory HDAC activity

**Figure 4.**
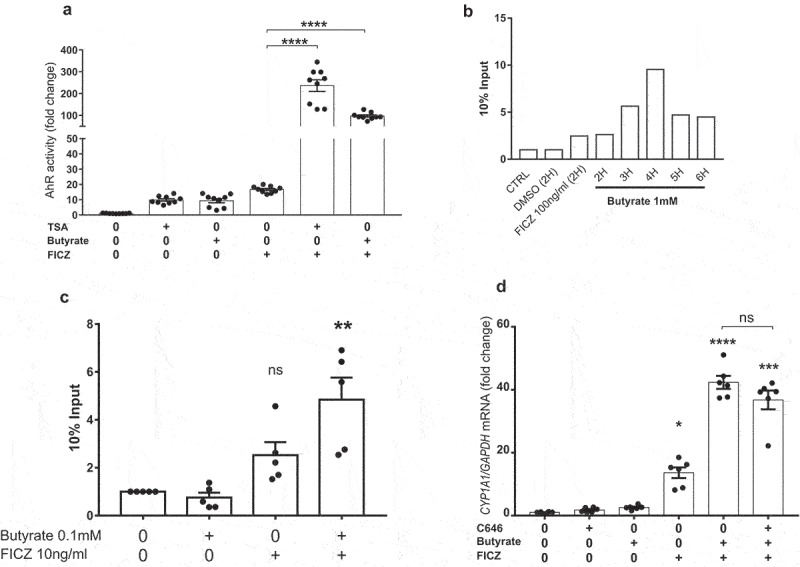
Butyrate acts through its iHDAC activity to activate AhR in synergie with AhR ligands. (a) AhR activity in HepG2^luc^ treated in triplicate with butyrate (1 mM), FICZ (10 ng/ml), TSA (100 nM) or the combination of FICZ with butyrate or TSA in a FBS-free media. Data are presented as mean ± SEM of three independent experiments. (b) AhR binding to the promoter region of the *CYP1A1* gene detected by ChIP-qPCR in HepG2 treated with DMSO, FICZ and butyrate at several time points. (c) Synergistic recruitment of AhR to the *CYP1A1* promoter mediated by butyrate in combination with FICZ. Only experiments with an enrichment greater than or equal to 1.5 for FICZ were retained. Data are presented as mean ± SEM and each dot represent an independent experiment. (d) RT-qPCR analysis of *CYP1A1* mRNA levels in HepG2 treated with butyrate (1 mM), FICZ (10 ng/ml), C646 (10 µM) or the combination of FICZ with butyrate or C646 for 8 hours. Data are presented as mean ± SEM of two independent experiment. **p < .01; ***p < .001; ****p < .0001.

Butyrate is a potent HDAC inhibitor (iHDAC), which mediates several of its biological effects.^29,30^ To explore the role of HDAC inhibition in the butyrate effect, we treated HepG2^luc^ cells with the iHDAC trichostatin A (TSA) in combination with FICZ. TSA recapitulated the effect of butyrate in enhancing FICZ-induced AhR activity, suggesting that butyrate acts through its iHDAC activity to activate AhR (Figure 4a). To confirm this result and precisely investigate whether chromatin conformational changes induced by butyrate mediate AhR recruitment at target genes, we performed a ChIP assay targeting the promoter of the *CYP1A1* gene. Treatment of HepG2 cells with butyrate resulted in a time-dependent increase in the recruitment of AhR with a peak at 4 H (Figure 4b). The combination of butyrate with FICZ induced an increase in the recruitment of AhR protein to the *CYP1A1* gene promoter (Figure 4c). To verify that the effect was not mediated by activation of the histone acetyltransferase (HAT) CBP/P300, cells were treated with C646, a HAT inhibitor, in combination with FICZ and butyrate. This combination did not lead to any change in the observed synergy (Figure 4d). Because induction of *CYP1A1* requires the presence of AhR, we evaluated whether iHDACs could act indirectly by increasing AhR expression. Although the 3 iHDACs tested (butyrate, TSA, and valproic acid (VPA)) showed a synergistic effect with butyrate in activating AhR (Figure S5A), only TSA significantly increased *AHR* expression (Figure S5B). Together, these results show that the increase in *CYP1A1* expression induced by iHDACs in combination with butyrate is not related to an increased *AHR* expression in HT29 cells. Butyrate synergizes with AhR agonists through its iHDAC activity by facilitating the access of the AhR complex to its binding sites in the promoter of target genes.

## Discussion

In this study, we provide new insight into the regulation of the AhR activation by microbial metabolites and endogenous ligands in the gut. While gut-derived butyrate activates AhR, we have shown that it does not bind to the receptor. Instead, it alters the chromatin conformation of the promoter of target genes by inhibiting HDAC to favor the binding of AhR-ligand complex and the activation, leading to synergistic effects between butyrate and microbiota-derived bona fide AhR agonists.

Metabolites produced by the gut microbiota play a fundamental role in the crosstalk between the microbiota and the host. The huge microbial diversity of microorganisms in the gut produces metabolites with various structural diversity and effects. Importantly, all these metabolites are present all together in the gut lumen, where they can interact with each other and with host cells.^31^ Several bacterial metabolites have previously been shown to activate AhR in vitro,^17^ but no studies have investigated their precise activity. The current study shows that the major SCFA butyrate synergizes with Trp-derived metabolites to activate AhR on intestinal and liver cells as well as on human intestinal tissues. We observed similar effects with other SCFAs propionate and acetate. However, we observed some differences in cell type-dependent activation, especially for acetate, which was more potent on the liver than intestinal cells. This seems logical as acetate is typically released into the bloodstream via the portal vein and reaches the liver, while butyrate is primarily absorbed and metabolized by the colonic epithelium.^32^

Butyrate has recently been suggested to be an AhR ligand according to in silico molecular modeling.^17^ However, using radio-ligand binding assay, we showed that neither butyrate nor propionate or acetate, bind to AhR. Moreover, our analysis did not show any characteristics of AhR ligands for butyrate (receptor binding, nuclear translocation and dimerization with ARNT),^33^ thus confirming that butyrate does not act as this way.

Several indirect mechanisms of activation exist for AhR. Class 1 CYPs degrade many drugs and some AhR ligands such as FICZ or benzo-α-pyrene.^22,24^ Several molecules have been shown to be CYP1A1 inhibitors allowing to block the degradation of potent AhR agonists like FICZ, and thus increasing AhR activation.^22,23,34,35^ Here, we showed in a cell-free assay that butyrate did not act as an inhibitor of CYP1A1 and that the synergy observed do not rely on it. Inhibition of AhR proteasomal degradation leads to a higher AhR pool in the cell and treatment with a proteasome inhibitor induces AhR activation.^27^ It was shown that butyrate is a moderate proteasome inhibitor,^28^ but in our hands, proteasome inhibitors did not mimic the effects of butyrate on AhR activation.

Butyrate act both on HDACs and HATs. We showed that treatment of cells with butyrate led to increased binding of the AhR protein to the promoter of the human *CYP1A1* target gene. Similar results were previously observed in mouse colonic cell line (YAMC),^36^ but not in both Caco2^36^ and Hepa-1 cells.^37^ These conflicting results could be explained by a higher FBS concentration (20%) used in Caco2 cells compared to YAMC cells (5% FBS only) and by a too long exposure time (16 H) for the experiments in Hepa-1 cells. Whereas these studies reported no increase in AhR recruitment at the *CYP1A1* promoter when butyrate is combined with carcinogenic high-affinity AhR ligands such as TCDD or benzo-α-pyrene,^36,37^ we found an increase in AhR recruitment at the *CYP1A1* promoter when butyrate was combined with endogenous AhR ligand FICZ. This difference could be explained by the nature of AhR ligand.

We showed that the iHDAC TSA recapitulates the synergistic effect of butyrate in the presence of an AhR ligand. The effect of iHDACs on the *AHR* promoter and the increase in AhR activity have been reported but, we showed that inhibition of HDAC by butyrate did not lead to activation of HATs, contrary to what has been published previously, and therefore that the synergy was only based on inhibition of HDAC.^38,39^

Butyrate showed a trend toward an increased *AHR* expression at 6 H. However, the stimulated expression of *CYP1A1* induced by butyrate in combination with FICZ is seen as early as 6 H. As the transcribed *AHR* gene must be first translated into the protein to bind to its ligand and then to the *CYP1A1* promoter, there is a temporal argument here that increased *AHR* expression cannot be involved in the increased *CYP1A1* expression induced by butyrate in combination with FICZ. Thus, iHDACs did not act indirectly by increasing *AHR* expression.

However, we cannot guarantee that butyrate acts only through promoter acetylation since many epigenetic modifications such as propionylation, butyrylation, and crotonylation rely on HDAC.^32^ Epigenetics has been gaining momentum for several years due to its involvement in gene dynamics. There are 18 HDAC enzymes and some are known to be involved in certain pathological phenomena. For example, *HDAC3* expression is reduced in intestinal epithelial cells of patients with IBD compared to healthy subjects,^40^ and mice lacking *HDAC3* in intestinal epithelial cells develop spontaneous inflammation.^40^

Besides butyrate and AhR agonists, a highly diverse and abundant amount of biologically active bacterial metabolites are found within the intestines. Future studies should focus on deciphering the interactions between different metabolites, especially those with epigenetic characteristics such as SCFAs.

In conclusion, we show that butyrate acts synergistically with endogenous microbiota-derived Trp ligands to increase AhR activity in mammals’ cells. Systematic analysis of all potential mechanisms involved revealed that butyrate is not an AhR ligand. However, butyrate potentiates AhR activation by increasing AhR recruitment to the target gene promoter by a mechanism dependent on the inhibition of HDAC. These results support the existence of complex interactions between multiple gut-derived metabolites that deserve extensive exploration. Moreover, it opens the way to new therapies to modulate AhR activities in the gut, using live biotherapeutics products producing butyrate and indoles, or by directly using mixtures of metabolites or via strong AhR ligands produced by microbial metabolite mimicry.^41^

## Materials and methods

### Cell culture and maintenance

Cell line HepG2 was obtained from the American Type Culture Collection (ATCC, Cat#HB-8065). Cells were maintained in EMEM (ATCC, Cat#30-2003) containing 10% fetal bovine serum (FBS) and 100 U/ml penicillin and streptomycin at 37°C in a 5% CO2 incubator. Cell line HT29 was obtained from the American Type Culture Collection (ATCC, Cat#HTB-38). Cells were maintained in DMEM (Gibco, Cat#41965) containing 10% FBS, 100 U/ml penicillin and streptomycin and 2 mM of glutamine at 37°C in a 10% CO2 incubator. HepG2-Lucia™ (Cat#hpgl-ahr) and HT29-Lucia™ (Cat#ht2l-ahr) AhR reporter cells were obtained from Invivogen. Cells were maintained according to the manufacturer protocol. Cell line LS180 was obtained from European Collection of Authenticated Cell Cultures (ECACC, Cat#87021202). Cells were maintained in DMEM (Sigma, Cat#D6546) supplemented with 10% (V/V) FBS, 2 mM L-glutamine and 1% (V/V) MEM non-essential amino acids (Sigma, Cat#M7145) in a 5% CO_2_ incubator.

According to the literature reporting a butyrate concentration of 10 mM in the human gut, we chose to use this dose to treat intestinal cells. We used lower doses on hepatic cells as butyrate is metabolized by intestinal cells, and the concentration reaching the liver is much lower. As the amount of butyrate that reaches the HDACs is unknown, we chose a lower concentration than the documented luminal or serum concentration.

### Human subjects

All individuals with colorectal cancer (age 20–79) were recruited in the Digestive Surgery Department of the Saint Antoine Hospital (APHP, Paris France), provided informed consent and were included in a prospective biobank “BiomHost”. Approval for human studies was obtained from the local ethics committee (Comité de Protection des Personnes Ile-de-France III, Am9098-3-3663-NI, on the 21/09/2019).

### Reagents and antibodies

Sodium butyrate (Cat#303410), sodium propionate (Cat#P5436), sodium acetate (Cat#S2889), FICZ (Cat#SML1489), indole acetic acid (Cat#I3750), glyceric acid (Cat#51738), N-acetyl aspartic acid (Cat#00920), valeric acid (Cat#240370), 3-phenyl propionic acid (Cat#W288918), fumaric acid (Cat#47910), succinic acid (Cat#14079), malic acid (Cat#240176), 2-oxoglutaric acid (Cat#75890), 3-(4-hydroxyphenyl)propionic acid (Cat#H52406), cholic acid (Cat#C6445), γ-aminobutyric acid (Cat#A2129), 2-aminobutyric acid (Cat#162663), 5-aminovaleric acid (Cat#123188), homoserine (Cat#H6515), ornithine (Cat#02375), 2,6 diaminopimelic acid (Cat#D1377), tyrosine methyl esther (Cat#T90808), taurine (Cat#T0625), tyramine (Cat#T90344), urocanic acid (Cat#859796), γ-butyrobetaine (Cat#403245), N-acetylglucosamine (Cat#A8625), pyridoxal (Cat#271748), pyridoxamine (Cat#P9158), pyridoxine (Cat#P5669), sarcosine (Cat#131776), 1,3-diaminopropane (Cat#D23602), cadaverine (Cat#D22606), glutaric acid (Cat#G3407), hydroxyindole (Cat#H31859), spermidine (Cat#S2626), 5-hydroxyindole acetic acid (Cat#H8876), indole (Cat#I3408), indole 3 lactic acid (Cat#I5508), indole 3 pyruvic acid (Cat#I7017), tryptamine (Cat#193747), tryptophol (Cat#T90301), indole 3 aldehyde (Cat#129445), indoxyl 3 sulfate (Cat#I3875), indole 3 carbinol (Cat#I7256), deoxycholic acid (Cat#30960), lithocholic acid (Cat#L6250), chenodeoxycholic acid (Cat#C9377), taurochenodeoxycholic acid (Cat#T6260), glycochenodeoxycholic acid (Cat#G0759), taurocholic acid (Cat#T40009), glycocholic acid (Cat#G1732), nicotinic acid (Cat#N4126), Mepenzolate bromide (Cat#M5651), MG132 (Cat#M8699), Trichostatine A (Cat#T8552) C646 (Cat#382113) and valproic acid (Cat#P4543) were purchased from Sigma Aldrich.

### Measurement of AhR activity

The AhR activity was measured using HepG2-Lucia™ AhR reporter cells (InvivoGen, France) and HT29-Lucia™ AhR reporter cells. Cells were seeded into a 96-well plate and stimulated with for 24 hours according to the manufacturer’s protocol. Luciferase activity was measured using a luminometer and Quanti-Luc reagent (InvivoGen). The results were normalized on the basis of the negative luciferase activity of the control and cytotoxicity measurement (CytoTox 96 Non-radioactive Cytotoxicity Assay, Promega).

### Tryptophan metabolites measurements

Trp and 20 Trp metabolites were quantified by liquid chromatography coupled with high-resolution mass spectrometry from FBS, murine and human serum as previously described.^42^

### Human intestinal explants treatment

Intestinal margins resection from patients undergoing surgery for colorectal cancer were obtained from the digestive surgery department of St Antoine Hospital. After collection, intestinal resection was opened longitudinally and washed in RPMI Glutamax (Gibco, Cat#61870-010) supplemented with 10% FBS, 50 µg/ml gentamicin (Sigma Aldrich, Cat#G1264) and 0.25 µg/ml amphotericine B (Sigma Aldrich, Cat#A2411). Mucosal samples (3 x 5 mm) were treated with several concentrations of butyrate and/or FICZ for 24 H at 37°C in a 5% CO_2_ incubator. Samples were placed in RNA later (Sigma Aldrich, Cat# R0901) for 48 H and frozen at −80°C before RNA extraction.

### Reverse transcription and quantitative polymerase chain reaction (RT-qPCR)

Total RNAs of cell lines were extracted with Trizol (Ambion, Cat#15596018). DNAse-treated RNAs were reverse transcribed with high capacity cDNA RT kit (ThermoFisher Scientific, Cat#4368814) for RT-qPCR. Specific cDNA were amplified for *CYP1A1* (Forward 5’-CAGCTCAGCTCAGTACCTC-3’; Reverse 5’- CTTGAGGCCCTGATTACCCA-3), *AHRR* (Forward 5’- GATGATGCTATCCTGGGGAGG −3’; Reverse 5’- CATCGTCATGAGTGGCTCG −3’), *HCAR2* (Forward 5’-TTCAGAGAATGCGATTTAGGG −3’; Reverse 5’- GAAGCAAAAGTTTCAGATGCC-3’) and *GAPDH* (Forward 5’- CAACGACCACTTTGTCAAGC-3’; Reverse 5’-TTCCTCTTGTGCTCTTGCTG-3’). qPCR assays were performed with SYBR Green PCR Master Mix (Applied Biosystems, Cat.No. 4309155) and carried out on a StepOne Plus (Applied Biosystems) running with StepOne Plus Software Relative quantification of *CYP1A1, AHRR* and *HCAR2* mRNA levels was expressed as fold-change, using the 2^−ΔΔCt^ method with *GAPDH* as reference gene.

### CYP1A1 inhibition assay

Activity of human recombinant CYP1A1 (Sigma Aldrich; Cat#C3735) was assessed using P450-Glo™ CYP1A1 Assay (Promega; Cat#V8751) and NADPH regeneration system (Promega; Cat#V9510). Reactions were performed in duplicate with butyrate concentrations ranging from 0.1 mM to 10 mM for 20 minutes. Trichostatine A (1 nM and 1 µM) was used as a positive control. Luminescence was recorded using a Spectra Max m5^e^ (Molecular devices), and a control without CYP1A1 was subtracted from each measurement to account for background. CYP1A1 activity was taken as the percentage of the luminescence after incubation with vehicle.

### Chromatin immunoprecipitation

HepG2 cells were seeded in a 6 well plate in EMEM (ATCC, Cat#30-2003). The following day, cells were incubated with DMEM, DMSO, FICZ (10 ng/ml), butyrate (1 mM), or the mixture of FICZ and butyrate (10 ng/ml and 1 mM respectively) for 180 minutes at 37°C. DNA-protein complexes were crosslinked by the addition of 80 µl 37% formaldehyde (Sigma Aldrich, Cat#252549) to 2 ml of media for 15 minutes at RT. Thereafter, 282 µl of 1 M glycine (Sigma Aldrich, Cat#G7126) was applied for 5 minutes at RT. The cells were collected and rinsed twice with ice-cold PBS 1X. Pellets were lysed in 1 ml of ice-cold ChIP buffer (NaCl 150 mM (Sigma Aldrich, Cat#S9888), Tris-HCl 50 mM; pH 7,5 (Sigma Aldrich, Cat#10812846001), EDTA 5 mM (Sigma Aldrich, Cat#EDS), NP-40 0.5% vol/vol (Sigma Aldrich, Cat#NP40S), Triton X-100 1% vol/vol (Sigma Aldrich, Cat#T8787), PMSF 0.5 mM (Sigma Aldrich, Cat#P-7626) and leupeptin 10 µg/ml (Sigma Aldrich, Cat#L-2884) followed by centrifugation (12,000 g for 5 minutes at 4°C). Pellets were rinced in 1 ml of ice-cold, followed by centrifugation (12,000 g for 5 minutes at 4°C). The resulting pellets were resuspended in 200 µl of ChIP buffer and sonicated for 20 cycles (30 sec ON – 30 sec OFF) in a Bioruptor Pico (Diagenode). Cellular debris were removed by centrifugation (12,000 g for 10 minutes at 4°C) and DNA concentration was determined in the supernatants using a Nanodrop 2000 (Thermo Fisher Scientific). An aliquot of 20 µg of chromatin was resuspended in a total volume of 800 µl of ChIP buffer. 20 µL was used for 10% Input and 200 µL was used for each immunoprecipitation (IP). IP was achieved by the addition of 5 µl of anti-AhR (D5S6H) antibody (Cell Signaling Technology, Cat#83200),1 µl of normal rabbit IgG (Cell Signaling Technology, Cat#2729) as the negative control and 1 µL of H3 (D2B12) antibody (Cell signaling technology, Cat#4620) as the positive control. Samples were incubated with rotation at 4°C overnight. The next day, ChIP-Grade protein G magnetic beads (Cell signaling technology, Cat#9006) were added and the samples were incubated for 2 hours at 4°C with rotation. Beads were briefly pelleted in magnetic separation rack Dynamag 2 (InvitroGen) and washed two times by 500 µl of ChIP buffer. The pellets were resuspended in 150 µl of ChIP buffer and boiled for 10 minutes at 100°C with 1300 rpm shaking in the Thermomixer Comfort (Eppendorf). 1 µl of proteinase K (20 µg/ml) was added and chromatin was incubated for 30 minutes at 55°C Thermomixer Comfort (Eppendorf). Samples were boiled again for 10 minutes at 100°C with 400 rpm shaking. After separation in the magnetic rack, chromatin was purified using NucleoSpin Gel and PCR Clean‑up kit (Machinerey-Nagel, Cat#740609) according to the manufacturer protocol and was used for quantitative PCR in SteponeOne Plus (Applied Biosystems). Then, 2 μl of DNA were used in the PCR reaction together with nuclease-free water, SYBR-Green PCR Master Mix (Applied Biosystems, Cat#4368814), and 5 µM *CYP1A1* promoter primers (5′-AGCTAGGCCATGCCAAAT-3′ and 5′-AAGGGTCTAGGTCTGCGTGT-3′) as described previously.^43^ The qPCR program was as follows: enzyme activation at 95°C for 3 minutes and denaturation with annealing and elongation for 40 cycles at 95°C for 15 seconds and at 60°C for 60 seconds for 40 cycles. Then, the 10% input method was applied and the results were expressed as fold enrichment next to non-treated sample.

### Radio-ligand binding assay

Cytosolic protein extracts from murine hepatoma Hepa1c1c7 cells (2 mg/mL) were incubated for 2 h at room temperature with 2 nM [^3^H]-TCDD in the presence of butyrate (0.1–50 mM), propionate (0.1–50 mM), acetate (0.1–50 mM), FICZ (100 nM; positive control), or vehicle (DMSO; 0.1% V/V; corresponds to specific binding of [^3^H]-TCDD = 100%). Additionally, combined incubation of protein extracts with 2 nM [^3^H]-TCDD, 10 mM butyrate and 10 nM FICZ was performed. Ligand binding to the cytosolic proteins was determined by the hydroxyapatite-binding protocol and scintillation counting as described elsewhere.^44^ Specific binding of [^3^H]-TCDD was determined as a difference between total and nonspecific (TCDF; 200 nM) reactions.

### Protein Co-immunoprecipitation of the AhR-ARNT

Colonic epithelial cell-line LS180 were treated with a vehicle (DMSO; 0.1% V/V), positive control (TCDD; 10 nM), butyrate (10 mM), FICZ (10 nM) and combination of butyrate (10 mM) and FICZ (10 nM) for 90 min. Cells were lysed and Pierce™ Co-Immunoprecipitation Kit (Thermo Fisher Scientific, Cat# 26149) with covalently bound AhR antibody (Santa Cruz Biotechnology, Cat#sc-133088) was used for co-immunoprecipitation of AhR-ARNT heterodimers. Eluted protein complexes, in parallel with parental total lysates, were resolved in SDS-PAGE gels followed by Western blot and immunodetection with ARNT 1 antibody (Santa Cruz Biotechnology, Cat#sc-17812). Chemiluminescent detection was performed using horseradish peroxidase-conjugated anti-mouse secondary antibody (Cell Signaling Technology, Cat#7076S) and WesternSure® PREMIUM Chemiluminescent Substrate (LI-COR Biotechnology) by C-DiGit® Blot Scanner (LI-COR Biotechnology). Subsequently, the blots were stripped using Re-Blot Plus Strong Solution (Millipore) and the AhR was immunodetected with AhR antibody and the same chemiluminescent procedure. The experiments were performed in two consecutive cell passages.

### Immunofluorescence detection of the AhR nuclear translocation

LS180 (90,000 cells/well) were grown on poly-D-lysine coated 8-well tissue culture chamber slides (Sarstedt) overnight. The cells were incubated with a vehicle (DMSO; 0.1% V/V), butyrate (10 mM), FICZ (10 nM) and combination of butyrate (10 mM) and FICZ (10 nM) for 90 min. After the treatment, the cells were washed with PBS, fixed with 4% (V/V) formaldehyde, permeabilized using 0.1% (V/V) Triton X-100, blocked with 3% (m/V) bovine serum albumin and incubated with Alexa Fluor 488 labeled primary antibody against AhR (Santa Cruz Biotechnology, Cat#sc-133088), as described elsewhere.^43^ Nuclei were stained with 4′,6-diamino-2-phenylindole (DAPI) and the slides were sealed by coverslips using VectaShield® Antifade Mounting Medium (Vector Laboratories). The AhR nuclear translocation was observed using Oympus Fluoview 1000 confocal system (Olympus); near UV laser (405 nm) for excitation of DAPI and Ar laser (488 nm) for excitation of Alexa Fluor 488. The experiments were performed in three consecutive cell passages. The level of AhR nuclear translocation was calculated as the proportion of fluorescence intensity of nucleus and fluorescence intensity of cytoplasm (fluorescence of nucleus/fluorescence of cytoplasm) and was expressed as a percentage of intensity of cytoplasm fluorescence. The level of nuclear translocation of AhR was estimated in 80–110 cells for every treatment in each experiment.

### Quantification and statistical analysis

Statistical analyses were performed using GraphPad Prism 7 software (www.graphpad.com). Mann Whitney test was used to compare two groups. Ordinary one-way ANOVA test was used to compare three groups. For ChIP experiment, One-way ANOVA corrected by the false discovery rate method of Benjamini and Hochberg was performed. P value of <0.05 was considered significant, *p < .05, **p < .01, ***p < .001, ****p < .0001

## Supplementary Material

Supplemental Material

## Data Availability

All data supporting the findings of this study are available within the paper and are available from the corresponding author upon request.
