# Supplementary figures and images for: Butyrate acts through HDAC inhibition to enhance aryl hydrocarbon receptor activation by gut microbiota-derived ligands
Source: Gut Microbes. 2022 Jul 27;14(1):2105637. doi: 10.1080/19490976.2022.2105637 (PMC9336500; doi:10.1080/19490976.2022.2105637)

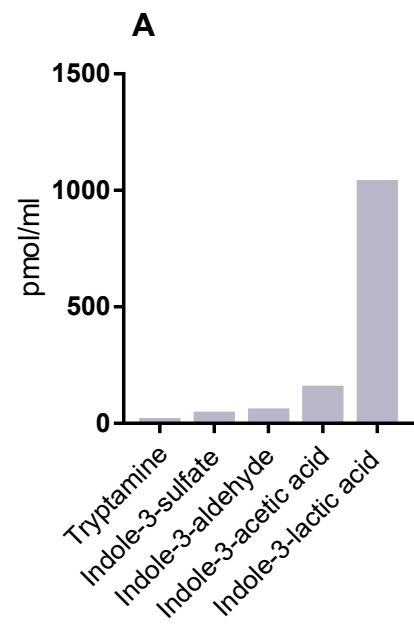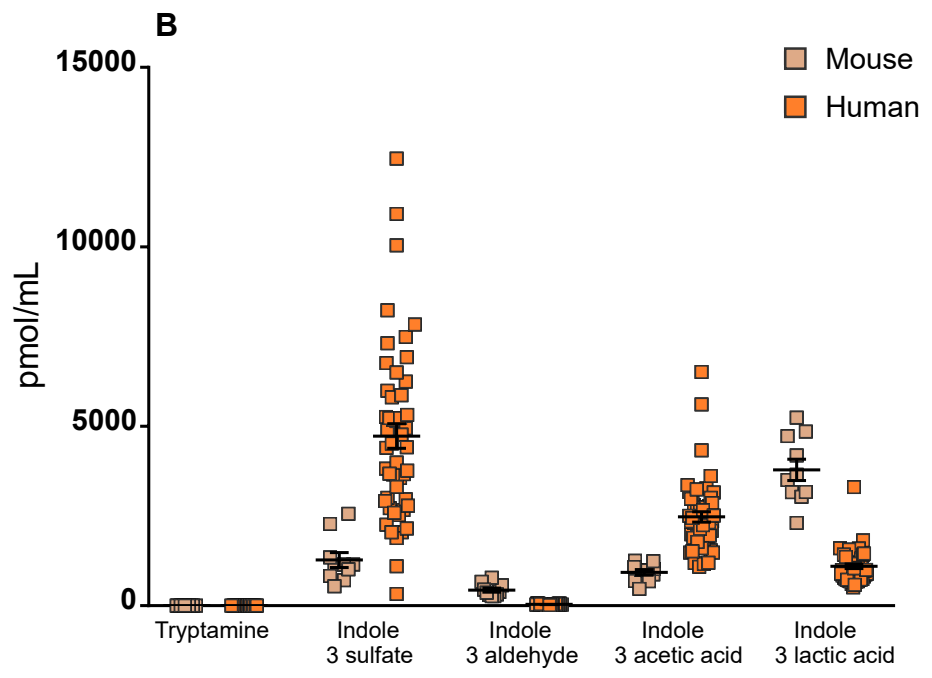

Supplement: Supplemental Material [file KGMI_A_2105637_SM2094.zip › Supp_Figure 1.pdf]

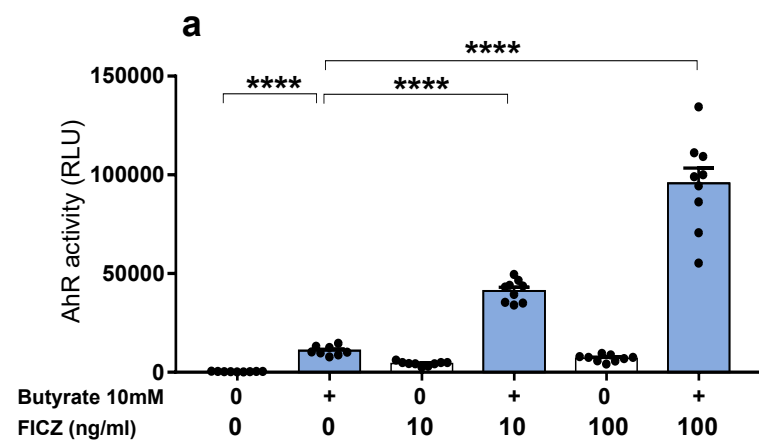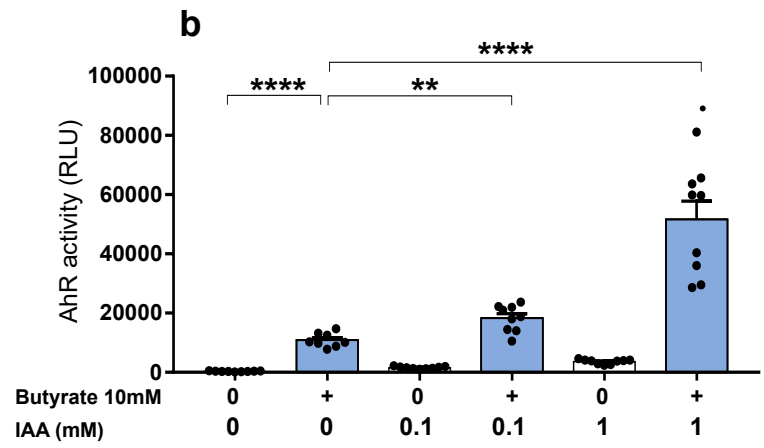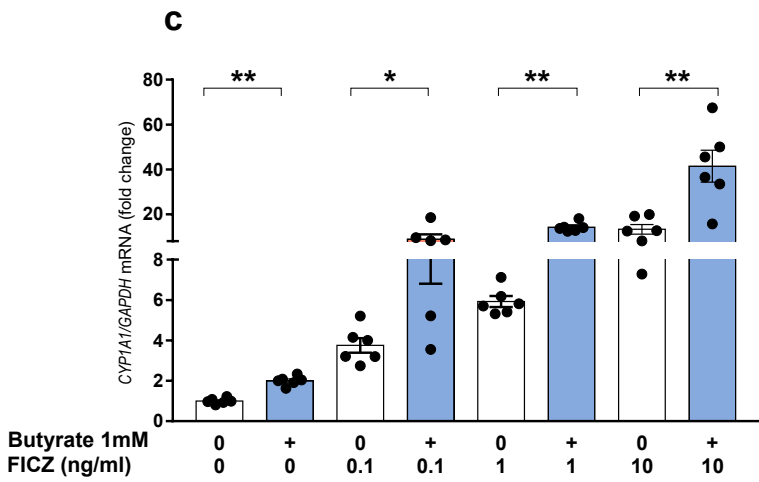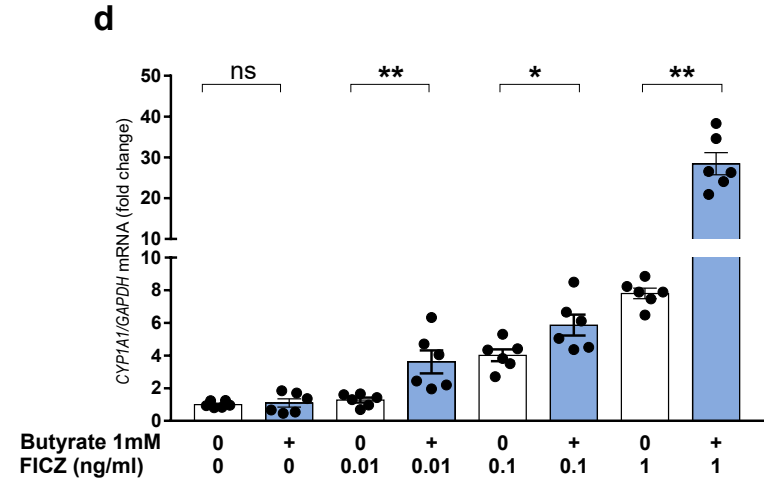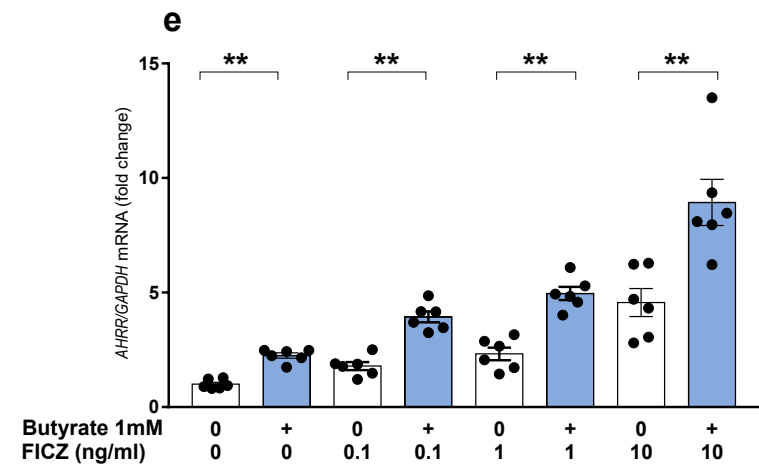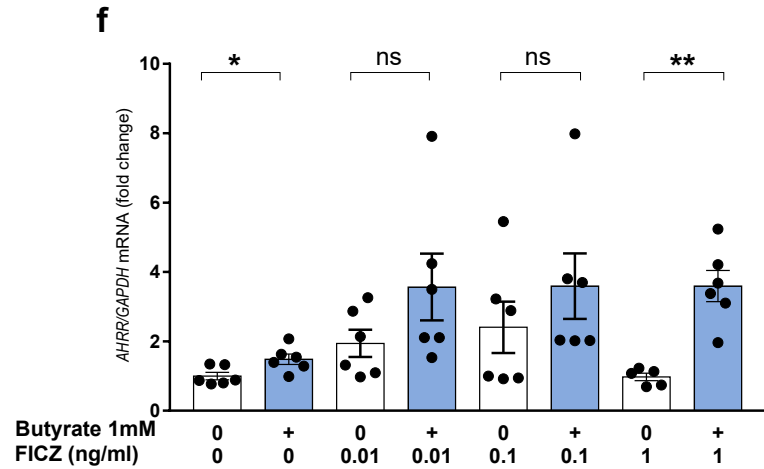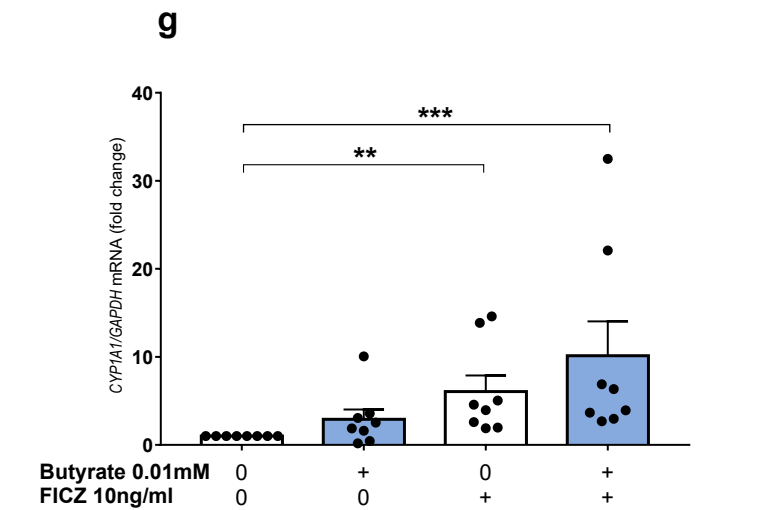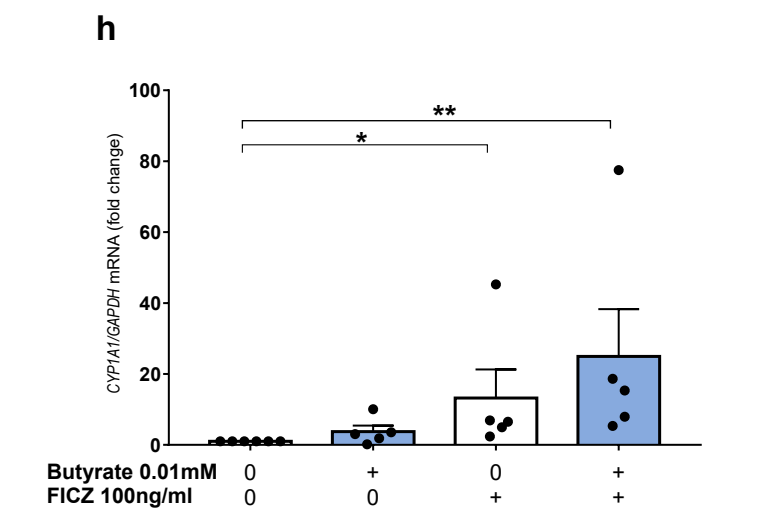

Supplement: Supplemental Material [file KGMI_A_2105637_SM2094.zip › Supp_Figure 2.pdf]

**a**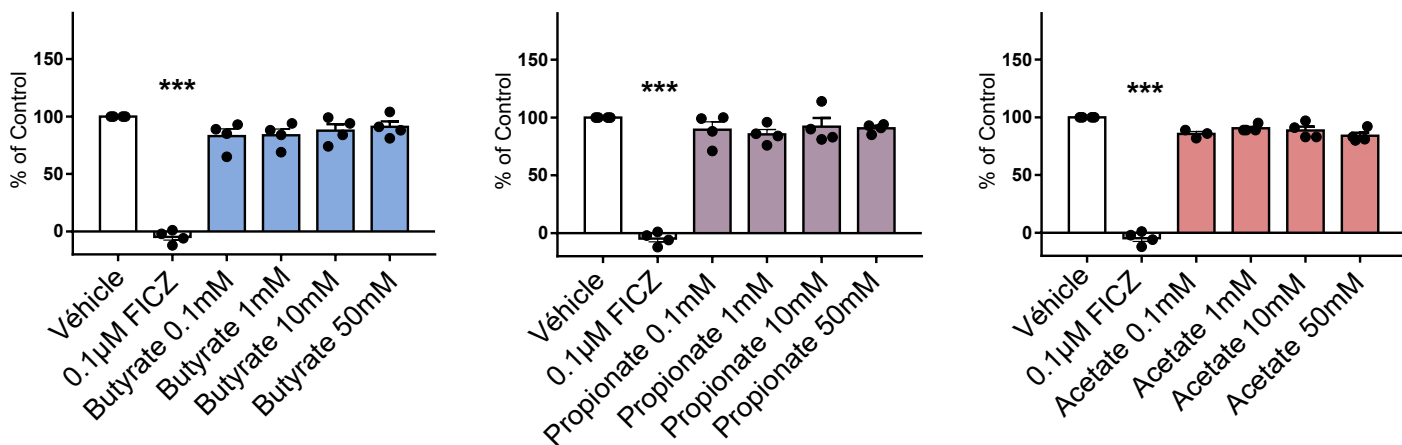**b**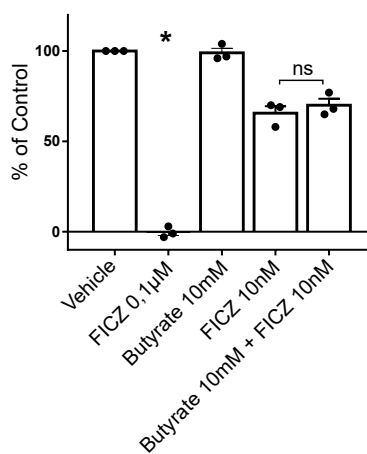**c**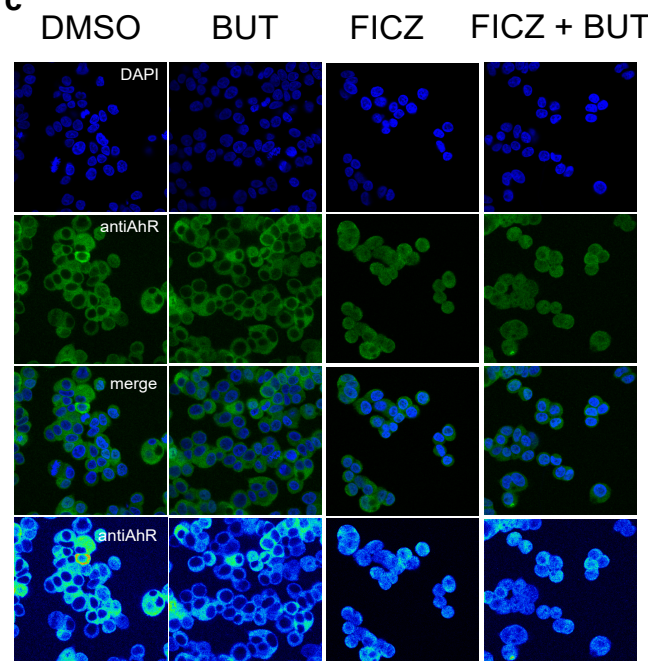**d**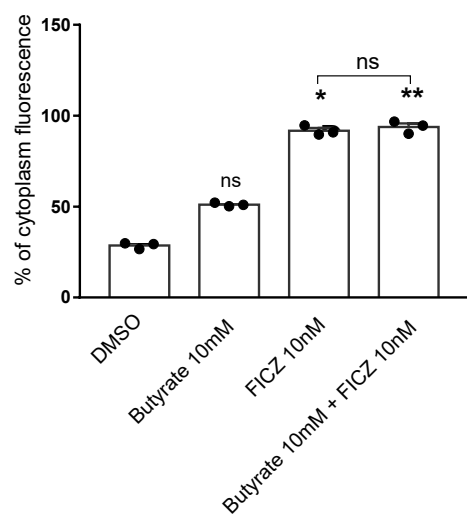**e**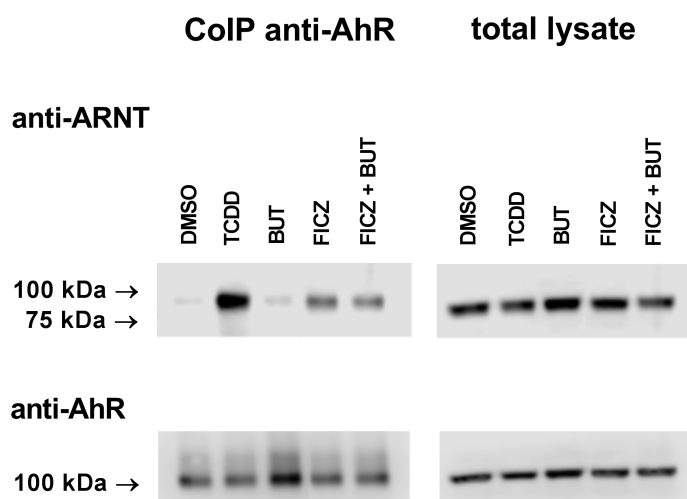

Supplement: Supplemental Material [file KGMI_A_2105637_SM2094.zip › Supp_Figure 3.pdf]

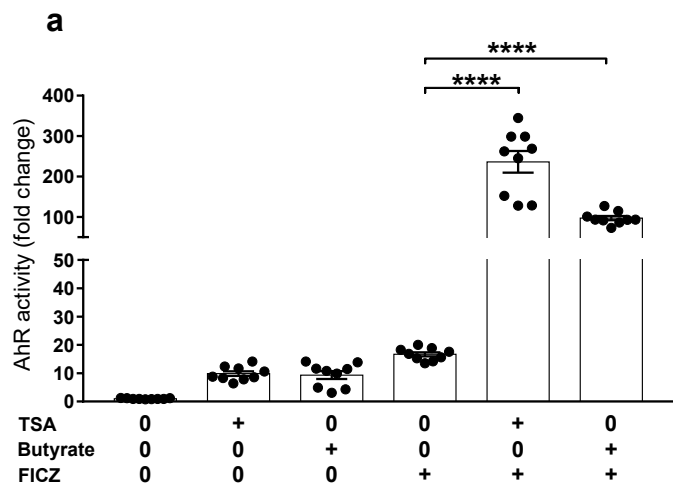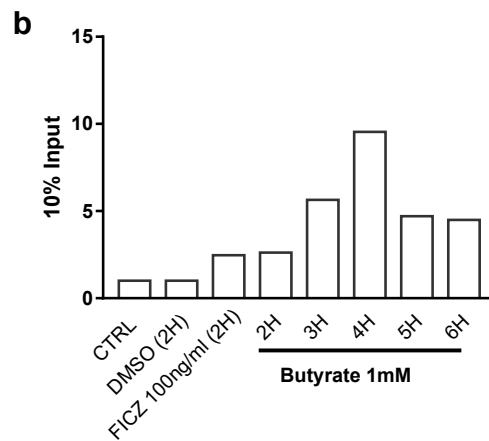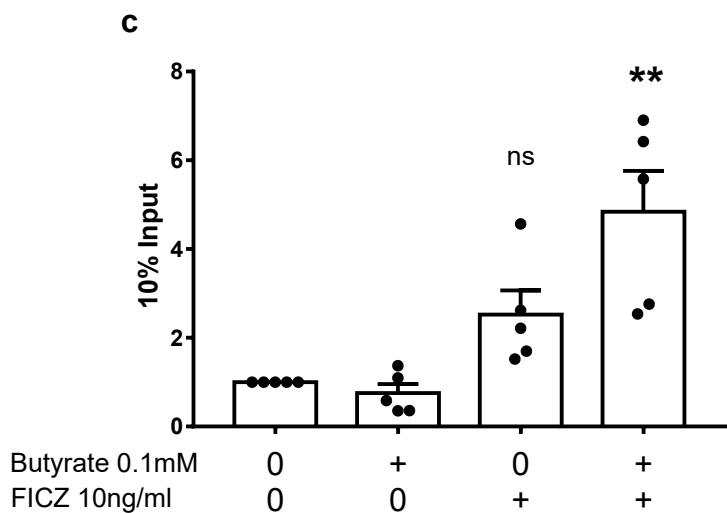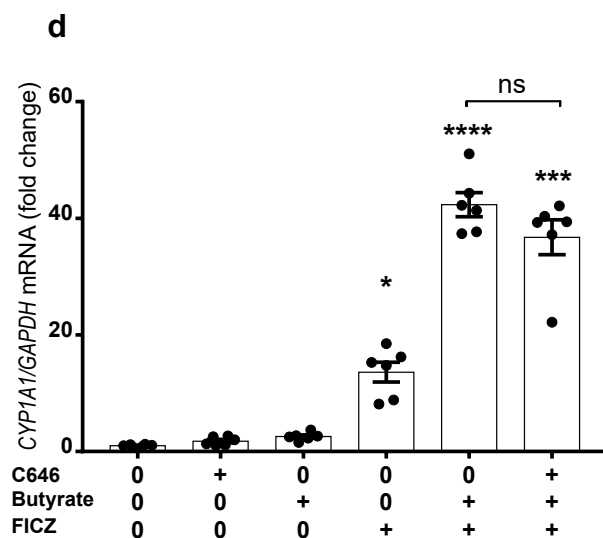

Supplement: Supplemental Material [file KGMI_A_2105637_SM2094.zip › Supp_Figure 4.pdf]

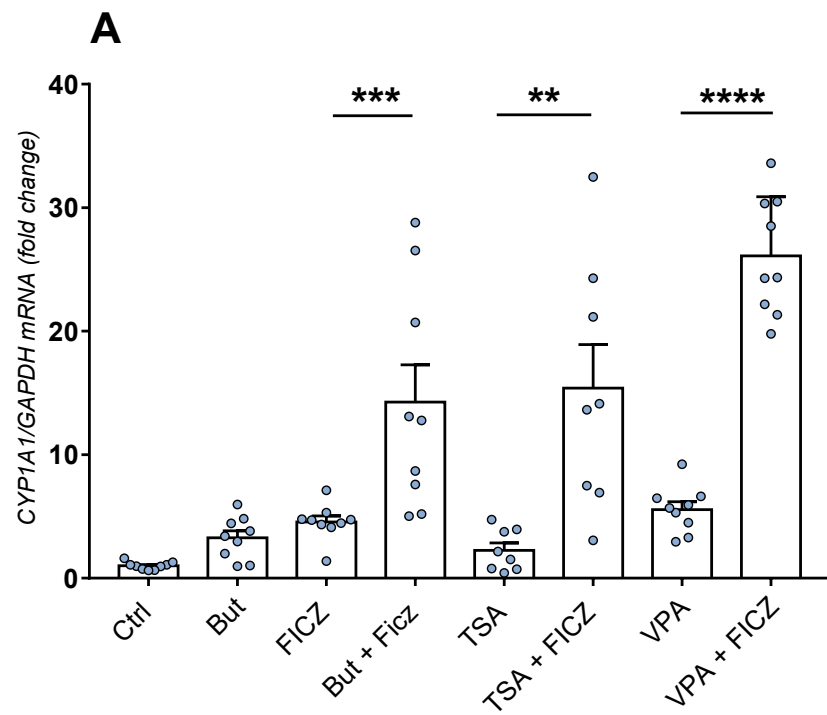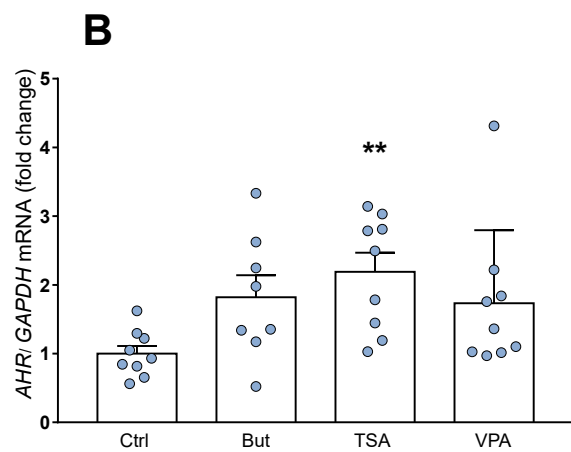

Supplement: Supplemental Material [file KGMI_A_2105637_SM2094.zip › Supp_Figure 5.pdf]
